# Antiretroviral treatment reverses HIV-associated anemia in rural Tanzania

**DOI:** 10.1186/1471-2334-11-190

**Published:** 2011-07-11

**Authors:** Asgeir Johannessen, Ezra Naman, Svein G Gundersen, Johan N Bruun

**Affiliations:** 1 Department of Infectious Diseases, Oslo University Hospital, Ulleval, Oslo, Norway; 2 HIV Care and Treatment Centre, Haydom Lutheran Hospital, Mbulu, Tanzania; 3 Research Unit, Sorlandet Hospital, Kristiansand, Norway; 4 Centre for Development Studies, University of Agder, Kristiansand, Norway; 5 Institute of Clinical Medicine, University of Tromso, Tromso, Norway; 6 Medical Department, University Hospital of North Norway, Tromso, Norway

## Abstract

**Background:**

HIV-associated anemia is common and associated with poor prognosis. However, its response to antiretroviral treatment (ART) in rural Africa is poorly understood.

**Methods:**

HIV-infected adults (≥15 years) who enrolled in HIV care at Haydom Lutheran Hospital in northern Tanzania were included in the study. The effect of ART (zidovudine/stavudine + lamivudine + efavirenz/nevirapine) on HIV-associated anemia was studied in a subset of patients who were anemic at the time they started ART and had a follow-up hemoglobin measurement 12 months later. Pregnant women were excluded from the study, as were women who had given birth within the past 6 weeks. Anemia was defined as hemoglobin <12 g/dL in women and <13 g/dL in men. We applied paired sample T-tests to compare hemoglobin levels before and one year after ART initiation, and logistic regression models to identify predictors of persistent anemia.

**Results:**

At enrollment, mean hemoglobin was 10.3 g/dL, and 649 of 838 patients (77.4%) were anemic. Of the anemic patients, 254 (39.1%) had microcytosis and hypochromia. Among 102 patients who were anemic at ART initiation and had a follow-up hemoglobin measurement after 12 months, the mean hemoglobin increased by 2.5 g/dL (*P *< 0.001); however, 39 patients (38.2%) were still anemic after 12 months of ART. Independent predictors of persistent anemia were mean cell volume in the lower quartile (<76.0 fL; Odds Ratio [OR] 4.34; 95% confidence interval [CI] 1.22-15.5) and a zidovudine-containing initial regimen (OR 2.91; 95% CI 1.03-8.19).

**Conclusions:**

Most patients had anemia at enrollment, of whom nearly 40% had microcytosis and hypochromia suggestive of iron deficiency. The mean hemoglobin increased significantly in patients who received ART, but one third were still anemic 12 months after ART initiation indicating that additional interventions to treat HIV-associated anemia in rural Africa might be warranted, particularly in patients with microcytosis and those treated with zidovudine.

## Background

Anemia is a common feature of HIV infection, occurring in approximately 35% of patients who initiate antiretroviral treatment (ART) in Europe and North America [1]. In addition to causing reduced physical functioning and quality of life, a number of studies have found that anemia at the time of ART initiation is associated with HIV disease progression and mortality [2-9]. Indeed, in the EuroSIDA cohort, patients with severe anemia at baseline had 13 times greater risk of death during the first year of ART than patients with normal hemoglobin levels [3]. Similar findings have recently been reported from Tanzania, Côte d'Ivoire, South Africa and Malawi [2,6,8].

It is uncertain whether the association between anemia and mortality is causal or whether anemia acts as a surrogate marker of underlying disease. Previous studies have found that the incidence of anemia increases with progression of HIV infection [9-11], and anemia is a known feature of certain opportunistic infections such as tuberculosis (TB), atypical mycobacteria and parvovirus B19 [12]. Several other etiologic factors may also be involved in the development of HIV-associated anemia, including micronutrient deficiencies, immunological myelosuppression, impaired erythropoietin production and blood loss from intestinal opportunistic disease.

Management of HIV-associated anemia in high-income countries includes erythropoietin treatment, which has been associated with recovery from anemia and improved survival, but high costs restrict its use in resource-limited settings [13]. Furthermore, studies from Europe and North America have shown that ART itself can be an effective treatment of the anemia of HIV infection [3,14-16]. However, it remains to be seen if the same holds true in rural Africa, where co-morbidities such as micronutrient deficiencies, malaria, TB, parasitic infections and genetic hematological disorders are common. Therefore, we aimed to characterize the anemia and cell indices in a cohort of HIV-infected adults in rural Tanzania, and to study the effect of ART and other factors on the hemoglobin evolution over time.

## Methods

### Study setting and participants

Our study was carried out in Tanzania, a low-income country in East Africa, with an estimated adult HIV prevalence of 5.7% [17]. Haydom Lutheran Hospital is a 400-bed hospital in Manyara region in northern Tanzania. The hospital is the main health care provider to a rural population of about 260,000 people, in an area where the majority rely on subsistence farming and pastoralism.

The HIV Care and Treatment Centre in Haydom has provided ART free of charge since October 2003. Clinical officers, under supervision of a physician, have been responsible for medical follow-up of patients. Most of the patients who enroll in the HIV program are diagnosed either through voluntary testing and counselling in the villages or testing of hospitalized patients with clinical suspicion of HIV/AIDS. Hence, many patients have advanced immunodeficiency at the time of enrollment.

In the present study we describe the baseline hematological profile in a cohort of treatment-naïve individuals aged 15 years or older who enrolled in HIV care at Haydom Lutheran Hospital between October 2003 and January 2008. In a subset of patients who were anemic at the time they started ART and had a follow-up hemoglobin measurement 12 months later we studied the effect of ART on HIV-associated anemia. Women who were pregnant at baseline or became pregnant during the observation period were excluded from the study, as were women who had given birth within the past 6 weeks, since the physiological hemodilution in pregnancy and subsequent blood loss during delivery would have precluded the results. Ethical approval was obtained from the Medical Research Coordinating Committee of the National Institute for Medical Research in Tanzania and Regional Committee for Medical Research Ethics in Norway. Patients gave written consent to participate in the study.

### Treatment, monitoring and laboratory investigations

ART was initiated in accordance with guidelines from the World Health Organization (WHO) and the National AIDS Control Program [18-21]: WHO stage 4 irrespective of CD4 cell count, WHO stage 3 with CD4 ≤350 cells/μL, or CD4 ≤200 cells/μL with any WHO stage. However, reliable CD4 cell counts were not available until September 2006, so most patients started ART based on clinical criteria only (WHO stage 3 or 4).

First-line treatment comprised stavudine or zidovudine, combined with lamivudine, and either nevirapine or efavirenz. Regimen choice was subject to availability, with use of a generic fixed-dose combination of stavudine, lamivudine and nevirapine whenever possible. Efavirenz was used in patients with concomitant TB treatment. None of the patients switched to second-line ART during the observation period. Patients with CD4 ≤200 cells/μL or WHO stage 3 or 4 disease got co-trimoxazole prophylaxis 960 mg thrice weekly or 480 mg daily. Patients were seen by a clinical officer every 3 months, and CD4 cell count and full blood count (including hemoglobin) was scheduled every 3-6 months as part of routine care.

HIV infection was established using two different rapid antibody tests, Determine HIV-1/2 (Abbott laboratories, Abbott Park, IL, USA) and Capillus HIV-1/2 (Trinity Biotech, Bray, Co Wicklow, Ireland), in accordance with recommendations from the National AIDS Control Program [21]. Standard hematology was measured using Sysmex KX-21 Hematology Analyzer (Sysmex Corp., Kobe, Japan). Body mass index (BMI, weight in kilograms divided by height in meters squared) was used to assess nutritional status. Body weight was measured at each clinic visit using the same manual scale, and height was measured using a stadiometer mounted on the scale.

Anemia was defined as a hemoglobin level <12 g/dL for women and <13 g/dL for men, in accordance with WHO guidelines [22]. We classified the anemia as mild (10-12 g/dL for women and 10-13 g/dL for men), moderate (8-10 g/dL) and severe (<8 g/dL). Microcytosis was defined as a mean cell volume (MCV) <80 fL and hypochromia as a mean corpuscular hemoglobin (MCH) <26 pg [23]. In patients who died, anemia was considered a contributing factor if the last hemoglobin before death was <6.5 g/dL [24], or if anemia was recorded as a death cause by the clinician in charge.

### Statistical analysis

In general, we applied the first available laboratory result as the enrollment value. If two values were obtained within a month, the mean was employed. In the study on the effect of ART on HIV-associated anemia, only patients who were anemic at the time they started ART and had a follow-up hemoglobin measurement 12 months later were considered. The laboratory result obtained closest to the start of ART was used as baseline, and this value had to be no more than 3 months prior to ART initiation. Laboratory results obtained during the first year on ART were rounded off to the nearest 3 months.

To avoid loss of power we attempted to avoid dichotomization of continuous variables, such as BMI, MCV and MCH [25]. Instead, BMI was categorized according to recognized cutpoints [26], whereas for MCV and MCH we used quartiles. Chi-square tests were used to study factors associated with anemia at enrollment. Paired samples T-tests were used to compare hemoglobin levels before and 12 months after ART initiation. Multivariable logistic regression analysis was used to identify predictors of persistent anemia after 12 months of ART. Clinical and laboratory variables were initially examined in bivariable models adjusting for baseline hemoglobin. CD4 cell counts were excluded because of too few observations. All variables with *P *< 0.2 in bivariable analyses were then entered in a multivariable model, using the forward stepwise (Wald) method. Multicollinearity was excluded using Spearman's correlation coefficient with a cutoff at 0.7. Data were analysed with SPSS version 16.0 for Windows (SPSS Inc., Chicago, Illinois, USA). All tests were two-sided and level of significance was set at *P *< 0.05.

## Results

### Patient characteristics at enrollment

**Figure 1:**
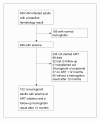
**Profile of the study cohort, Haydom Lutheran Hospital, Tanzania**.

A total of 838 HIV-infected adults who enrolled in the HIV program between October 3, 2003, and January 31, 2008, were included in this study (Figure 1). The mean age was 37 years (standard deviation [SD] 10) and 545 patients (65.0%) were women. Mean BMI was 18.6 kg/m^2 ^(SD 3.4). At enrollment, 399 patients (47.6%) had clinical AIDS (WHO stage 4), 245 (29.2%) had WHO stage 3, 97 (11.6%) had WHO stage 2, and 97 (11.6%) had WHO stage 1 disease. Ninety-eight patients (11.7%) were on TB treatment when they enrolled in care.

The mean hemoglobin at enrollment was 10.3 g/dL (SD 2.4), the mean MCV was 82.5 fL (SD 7.8), and the mean MCH was 26.6 pg (SD 3.2). Overall, 649 of 838 patients (77.4%) met the definition of anemia: 269 patients (32.1%) had mild anemia, 238 (28.4%) had moderate anemia, and 142 (16.9%) had severe anemia. Out of 649 anemic patients, 254 (39.1%) had microcytosis and hypochromia, suggestive of iron deficiency, whereas 52 (8.0%) had hypochromia with normocytosis, and 18 (2.8%) had microcytosis with normochromia.

Table 1 gives an overview of patient characteristics at enrollment and associations with anemia. Female gender, clinical AIDS, TB, low BMI, microcytosis and hypochromia were all significantly associated with anemia at enrollment.

**Table 1:** Patient characteristics and degree of anemia at enrollment

Variables	Normal n (%)	**Mild anemia**^**a**^ n (%)	**Moderate anemia**^**b**^ n (%)	**Severe anemia**^**c**^ n (%)	*P*
All (*n *= 838)	189 (22.6)	269 (32.1)	238 (28.4)	142 (16.9)	
Gender					0.046
Female	115 (21.1)	165 (30.3)	171 (31.4)	94 (17.2)	
Male	74 (25.2)	104 (35.5)	67 (22.9)	48 (16.4)	
Age (years)					0.726
<30	40 (20.1)	61 (30.7)	61 (30.7)	37 (18.6)	
30-39	75 (22.1)	106 (31.3)	98 (28.9)	60 (17.7)	
≥40	74 (24.7)	102 (34.0)	79 (26.3)	45 (15.0)	
AIDS^d^					<0.001
Yes	48 (12.0)	109 (27.3)	146 (36.6)	96 (24.1)	
No	141 (32.1)	160 (36.4)	92 (21.0)	46 (10.5)	
Tuberculosis^e^					0.001
Yes	9 (9.2)	34 (34.7)	28 (28.6)	27 (27.5)	
No	180 (24.3)	235 (31.8)	210 (28.4)	115 (15.5)	
BMI (kg/m2)^f^					<0.001
<16	17 (8.8)	49 (25.4)	67 (34.7)	60 (31.1)	
16-18.4	46 (19.7)	74 (31.8)	76 (32.6)	37 (15.9)	
≥18.5	118 (33.5)	134 (38.1)	76 (21.6)	24 (6.8)	
MCV (fL)					<0.001
<80	36 (11.7)	90 (29.2)	116 (37.7)	66 (21.4)	
≥80	153 (28.9)	179 (33.8)	122 (23.0)	76 (14.3)	
MCH (pg)					<0.001
<26	35 (10.3)	95 (27.9)	130 (38.1)	81 (23.7)	
≥26	154 (31.0)	174 (35.0)	108 (21.7)	61 (12.3)	

^a^Hemoglobin 10-12 g/dL (10-13 g/dL for men). ^b^Hemoglobin 8-10 g/dL. ^c^Hemoglobin <8 g/dL. ^d^World Health Organization clinical stage 4. ^e^On tuberculosis treatment at enrollment. ^f^60 values missing (*n *= 778).

BMI, body mass index; MCV, mean cell volume; MCH, mean corpuscular hemoglobin.

### Hemoglobin evolution during antiretroviral treatment

Out of 838 adults included in the study, 102 patients who were anemic at ART initiation and had a follow-up hemoglobin measurement 12 months later were selected for the sub-study on ART and anemia. Reasons for not being included in this sub-study are given in figure 1.

Among 102 patients in this sub-study, the mean age was 36 years (SD 10) and 75 patients (73.5%) were women. Mean BMI was 18.2 kg/m^2 ^(SD 3.3). Sixty-one patients (59.8%) had clinical AIDS at ART initiation. Fourteen patients (13.7%) were on TB treatment when they initiated ART and 8 more started TB treatment during the observation period of 12 months. Initial ART regimen was stavudine/lamivudine/nevirapine in 62 patients (60.8%), stavudine/lamivudine/efavirenz in 17 (16.7%), zidovudine/lamivudine/nevirapine in 16 (15.7%), and zidovudine/lamivudine/efavirenz in 7 (6.9%). Only 17 patients had a baseline CD4 measurement; the mean CD4 cell count was 140 cells/μL (SD 149).

**Figure 2:**
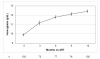
**Mean hemoglobin at baseline and after receiving antiretroviral treatment for 3, 6, 9 and 12 months**. Only patients with anemia at baseline and a follow-up hemoglobin after 12 months are presented (*n *= 102). Vertical bars indicate standard errors of the mean.

At the time of ART initiation the mean hemoglobin was 9.9 g/dL (SD 1.5). Eleven patients (10.8%) had severe anemia, 37 (36.3%) had moderate anemia and 54 (52.9%) had mild anemia. Patients with severe anemia were less likely to start a zidovudine-containing initial regimen (1 of 11; 9.1%) than patients with moderate (8 of 37; 21.6%) or mild anemia (14 of 54; 25.9%), but the difference was not statistically significant (*P *= 0.470). After receiving ART for 12 months the mean hemoglobin increased to 12.4 g/dL (SD 1.9) (*P *< 0.001) (Figure 2); however, 39 patients (38.2%) were still anemic, of whom 2 (2.0%) had severe anemia, 7 (6.9%) had moderate anemia and 30 (29.4%) had mild anemia. The two patients with severe anemia at 12 months both had a falling hemoglobin level during ART. The greatest hemoglobin change (+4.6 g/dL) was seen in patients with severe anemia at the time of ART initiation, compared to +3.0 g/dL in patients with moderate anemia and +1.8 g/dL in patients with mild anemia.

To assess whether the hemoglobin increase could be a result of other factors associated with enrollment in HIV care rather than ART itself, we studied the hemoglobin evolution in 18 non-pregnant adults who were anemic at enrollment and had a follow-up hemoglobin measurement 12 months later, but did not start ART. In this group there was no statistically significant hemoglobin increase: the mean hemoglobin was 10.1 g/dL (SD 1.5) at enrollment and 10.9 g/dL (SD 2.1) after 12 months (*P *= 0.191). Adjusted for baseline hemoglobin, patients who did not start ART had a 6-fold increased risk of persistent anemia after 12 months compared to those who started ART (Odds Ratio [OR] 5.99; 95% confidence interval [CI] 1.82-19.8; *P *= 0.003).

Sixty-five patients who were alive and in care 12 months after ART initiation, but who did not have a follow-up hemoglobin measurement, were excluded from our analysis. These patients had a similar age (mean 38 years) and sex (67.7% female) distribution as in the main analysis. Forty-one of these patients had a hemoglobin measurement at 9 months, and they showed a similar improvement as patients in the main analysis, from a mean hemoglobin of 9.6 g/dL (SD 1.8) at baseline to 12.3 g/dL (SD 1.6) at 9 months (*P *< 0.001).

Ninety-eight patients who died during the first year on ART were also excluded from our analysis, 68 of whom died within the first three months. At the time of ART initiation the mean hemoglobin among patients who died was 8.8 g/dL (SD 1.7), significantly lower than among patients in the main analysis (*P *< 0.001). We reviewed the records of these patients and found that anemia contributed to the death in 15 of 98 patients. Thirteen of 15 anemia-related deaths occurred in patients who received a stavudine-containing regimen, of whom 8 had severe anemia already at the time of ART initiation. Two patients died of presumed hematotoxic effects of zidovudine, 58 and 94 days after ART initiation, respectively.

### Predictors of persistent anemia

Among patients who were anemic at ART initiation, only MCV in the lower quartile (<76.0 fL) significantly predicted persistent anemia 12 months later in bivariable logistic regression analysis adjusted for baseline hemoglobin, whereas MCH in the lower quartile (<23.8 pg) and a zidovudine-containing initial regimen were borderline significant (Table 2). MCV and MCH were collinear (Spearman's correlation coefficient 0.86); hence, only MCV was included in the multivariable model. In the final multivariable analysis, MCV in the lower quartile (OR 4.34; 95% CI 1.22-15.5; *P *= 0.023) and a zidovudine-containing initial regimen (OR 2.91; 95% CI 1.03-8.19; *P *= 0.043) were significantly associated with persistent anemia.

**Table 2:** Predictors of persistent anemia after 12 months of ART among 102 HIV-infected adults who were anemic at ART initiation

			**Crude**^**a**^		**Adjusted**^**b**^	
				
Variables	Total	Anemic after 12 months (%)	OR (95% CI)	*P*	OR (95% CI)	*P*
Gender						
Female	75	30 (40.0)	1			
Male	27	9 (33.3)	0.85 (0.33-2.20)	0.744		
Age (years)						
<30	26	10 (38.5)	1			
30-39	42	19 (45.2)	1.45 (0.52-4.01)	0.476		
≥40	34	10 (29.4)	0.77 (0.25-2.33)	0.641		
AIDS^c^						
No	41	16 (39.0)	1			
Yes	61	23 (37.7)	0.93 (0.41-2.13)	0.862		
Tuberculosis^d^						
No	80	31 (38.8)	1			
Yes	22	8 (36.4)	0.69 (0.24-1.94)	0.477		
BMI (kg/m^2^)						
<16	26	9 (34.6)	1			
16-18.4	35	15 (42.9)	1.62 (0.55-4.78)	0.382		
≥18.5	40	14 (35.0)	1.24 (0.42-3.64)	0.702		
MCV (fL)						
≥87.5	25	6 (24.0)	1		1	
82.1-87.4	27	9 (33.3)	1.47 (0.43-5.06)	0.538	1.35 (0.38-4.72)	0.644
76.0-82.0	25	9 (36.0)	1.75 (0.51-6.00)	0.377	1.42 (0.39-5.13)	0.595
<76.0	25	15 (60.0)	4.05 (1.17-14.1)	0.028	4.34 (1.22-15.5)	0.023
MCH (pg)						
≥28.1	26	7 (26.9)	1			
26.6-28.0	24	7 (29.2)	1.14 (0.33-3.95)	0.841		
23.8-26.5	26	10 (38.5)	1.70 (0.52-5.52)	0.380		
<23.8	26	15 (57.7)	3.20 (0.97-10.5)	0.056		
Initial regimen						
Stavudine	79	27 (34.2)	1		1	
Zidovudine	23	12 (52.2)	2.46 (0.93-6.54)	0.071	2.91 (1.03-8.19)	0.043

^a^Adjusted for baseline hemoglobin. ^b^Adjusted for baseline hemoglobin and the other variables listed in the final model. MCH was excluded because of collinearity with MCV. ^c^World Health Organization clinical stage 4. ^d^On tuberculosis treatment at ART initiation or started within the observation period of 12 months.

OR, odds ratio; CI, confidence interval; BMI, body mass index; MCV, mean cell volume; MCH, mean corpuscular hemoglobin.

During the first year on ART, 3 patients switched from stavudine to zidovudine and 4 from zidovudine to stavudine, 2 of whom switched because of severe anemia. To assess whether drug substitutions might have confounded our results, we conducted a sensitivity analysis where these patients were excluded. In the resulting multivariable model, both MCV in the lower quartile (OR 4.30; 95% CI 1.16-15.9; *P *= 0.029) and a zidovudine-containing initial regimen (OR 3.32; 95% CI 1.10-10.0; *P *= 0.034) remained strong and significant predictors of persistent anemia.

## Discussion

In this study 77.4% of HIV-infected adults had anemia at the time they enrolled in HIV care. Women were more likely than men to be anemic, reflecting the overall higher prevalence of anemia in women due to menstrual blood loss and multiple deliveries. Furthermore, anemia at enrollment was associated with advanced immunodeficiency (WHO stage 4 and low BMI) and TB, in line with previous studies [9-11,27,28]. The overall prevalence of anemia was much higher in our study than in studies from high-income countries, but comparable to results from certain other African countries such as Nigeria, Côte d'Ivoire, Malawi and South Africa [2,29,30], probably because of more advanced immunodeficiency at enrollment as well as higher prevalence of anemia in the general population in sub-Saharan Africa [22]. Interestingly, in a recent study from our study site the mean hemoglobin among HIV-negative healthy adults (58.8% females, mean age 32.6 years) was 13.2 g/dL, compared to 10.3 g/dL among HIV-positive adults in our study [31]. In other words, anemia is far more frequent in HIV-infected adults in this region than in their HIV-negative counterparts.

The hemoglobin level increased significantly in patients who received ART; on average the hemoglobin increased 2.5 g/dL over the first 12 months among patients who were anemic at ART initiation. Previous studies have reported that ART is associated with resolution of HIV-associated anemia in Europe and North America [3,14,15]. More recently, similar findings have been reported from sub-Saharan Africa. In a study from Abidjan, Côte d'Ivoire, the prevalence of anemia (hemoglobin <10.5 g/dL) decreased from 31% at baseline to 17% after 6 months on a zidovudine-containing ART regimen [32]. A study from rural Uganda found that the mean hemoglobin increased from 11.3 g/dL at baseline to 12.8 g/dL after 12 months on ART [33]. Finally, a recent study from Kampala, Uganda, reported that the median hemoglobin increased by 2.9 g/dL during the first 6 months of ART among patients who were anemic (hemoglobin <9.5 g/dL) at ART initiation [34]. Although the magnitude of the hemoglobin increase might vary depending on the baseline hemoglobin level and degree of immunodeficiency, our study confirms that HIV-associated anemia in rural Tanzania, as in industrialized countries, can be reversed by ART in the majority of subjects. This suggests that HIV-associated anemia in this setting is caused mainly by factors related to the HIV infection itself, such as chronic inflammation and opportunistic infections, rather than tropical diseases or specific environmental factors.

However, not all patients on ART achieved a normal hemoglobin level. A low MCV was a strong and independent predictor of persistent anemia in our study. Microcytosis was also associated with persistent anemia in the Women's Interagency HIV Study in USA [15], and with an increased risk of developing severe anemia in a recent study from Uganda [34]. Classically, the most common cause of microcytosis is iron deficiency [35], which would explain why these patients were less likely to recover from anemia. On the other hand, iron supplementation in HIV is controversial, as there is evidence of iron accumulation and enhanced oxidative stress with progression of HIV infection [36]. Interestingly, in a study from Malawi only 16% of patients with severe HIV-associated anemia (hemoglobin <7 g/dL) had iron deficiency, whereas 19% had iron excess, assessed by bone marrow examination [37]. Moreover, a large study from Gambia found that elevated iron status strongly predicted mortality in HIV-infected adults [38]. In sub-Saharan Africa iron supplementation is commonly prescribed or self-supplemented; hence, there is a need for further studies to clarify its role in patients with HIV-associated anemia.

An initial zidovudine-containing ART regimen was also associated with persistent anemia in our study, increasing the risk by nearly 3 times compared to regimens containing stavudine. The potential myelosuppressive effect of zidovudine has been known for more than two decades [39], but its significance in clinical practice is still debated. For instance, a recent study from Uganda did not find an increased risk of early severe anemia (hemoglobin ≤8 g/dL within 6 months of ART initiation) in patients on zidovudine, and concluded that zidovudine - in the absence of better alternatives - should not be withheld even in patient with a hemoglobin below 8 g/dL at the time of ART initiation. However, the same study reported that anemic patients (hemoglobin ≤9.5 g/dL) who started a stavudine-containing regimen had a significantly larger hemoglobin increase than those who started zidovudine (3.1 g/dL vs. 2.5 g/dL) [34]. This is in line with a meta-analysis of 6 randomized trials, in which the mean hemoglobin level was 0.8 g/dL lower in patients who received zidovudine than those who received stavudine after 48 weeks on ART [40]. These differences are small, but the EuroSIDA study indicated that even subtle differences in hemoglobin level might be of clinical significance; indeed, a 1 g/dL decrease in the latest hemoglobin level increased the hazard of death by 57%, after adjusting for demographic factors, ART regimen, AIDS status, CD4 cell count and viral load [3]. The 2010 revision of the WHO guidelines for resource-limited settings recommend to start ART with a regimen consisting of zidovudine or tenofovir in combination with lamivudine and a non-nucleoside reverse transcriptase inhibitor, in order to avoid the long-term toxicities of stavudine [24]. Our study underscores the possible caveats of zidovudine, and suggests that other drugs might be preferable in anemic patients, especially in countries where anemia is endemic.

Before the HIV era, recommendations for management of anemia in classical tropical medicine included screening for malaria, treatment for helminthic infections and iron/folic acid supplementation [41]. In the setting of a generalized HIV epidemic, which is the case in most of sub-Saharan Africa, however, these guidelines are overdue for revision, since HIV-associated anemia requires a different approach. Indeed, in a study of patients admitted at a large urban hospital in Malawi with severe anemia (hemoglobin <7 g/dL), 79% had HIV and 37% had TB, and relatively few had classical causes of anemia such as heavy hookworm infections (9%), malaria (14%) or iron deficiency (25%) [37]. Management of anemia in settings with a high prevalence of HIV should always include an HIV test, and ART should be initiated if no other explanation of the anemia can be identified. Our study suggests that this would be sufficient to correct the anemia in the majority of subjects, and that MCV can be a useful additional test to identify patients in whom additional treatment (such as iron supplementation) might be required.

There were some limitations of our study. Most importantly there may have been a selection bias towards patients with a more favorable prognosis in the sub-study on the effect of ART on anemia, since we only included patients with a follow-up hemoglobin measurement after 12 months. Patients who died during the first year on ART might have had a different hemoglobin evolution, but since the majority of deaths occurred within the first 3 months, few of these had a second laboratory measurement. We also excluded patients who were still receiving ART, but did not have a hemoglobin measurement after 12 months. If these patients were less likely to be anemic than the others, our results could be biased (selection by indication); however, patients who were excluded had a similar hemoglobin increase at 9 months as the patients in our main analysis. Furthermore, our study was limited by lack of adherence data, and it is possible that poor adherence contributed to persistent anemia in some patients. On the other hand, in a previous study from the same cohort, we found that 94.8% of patients had suppressed viraemia after 1 year of ART, indicating good adherence in the majority of subjects [42]. Finally, given the observational nature of the study, the lack of randomization made comparison between different ART regimens susceptible to bias. For instance, patients with severe anemia at baseline were less likely to receive a zidovudine-containing regimen; however, we adjusted for baseline hemoglobin in the logistic regression models. Nevertheless, our results may have been influenced by other confounding factors and bias not accounted for in the analysis.

## Conclusions

In conclusion, we found that the majority of patients who enrolled in HIV care at a rural hospital in Tanzania were anemic, and that nearly 40% of the anemic patients had microcytosis and hypochromia suggestive of iron deficiency. Patients who were anemic at the time of ART initiation had a strong and significant hemoglobin increase over the initial 12 months of ART. However, more than one third of patients did not reach normal hemoglobin levels while on ART, and a low MCV and a zidovudine-containing initial regimen were independent predictors of persistent anemia. Our study indicate that although ART effectively reverses HIV-associated anemia in the majority of individuals, additional interventions might be warranted in a subset of patients.

## Competing interests

The authors declare that they have no competing interests.

## Authors' contributions

AJ analyzed the data and drafted the manuscript. EN collected the data. SGG and JNB conceived the study and participated in its design and coordination. All authors critically revised the manuscript and approved the final version.

## Pre-publication history

The pre-publication history for this paper can be accessed here:

http://www.biomedcentral.com/1471-2334/11/190/prepub
